# Potential advantages of genetically modified mesenchymal stem cells in the treatment of acute and chronic liver diseases

**DOI:** 10.1186/s13287-023-03364-x

**Published:** 2023-05-24

**Authors:** Farnaz Sani, Mahsa Sani, Zahra Moayedfard, Maryam Darayee, Lobat Tayebi, Negar Azarpira

**Affiliations:** 1 Hematology and Cell Therapy Department, Faculty of Medical Sciences, Tarbiat Modares University, Tehran, Iran; 2 Department of Tissue Engineering and Cell Therapy, School of Advanced Technologies in Medicine, Shiraz University of Medical Sciences, Shiraz, Iran; 3 Department of Molecular Medicine, School of Advanced Medical Sciences and Technologies, Shiraz University of Medical Sciences, Shiraz, Iran; 4 Marquette University School of Dentistry, Milwaukee, WI 53233 USA; 5 Transplant Research Center, Shiraz University of Medical Sciences, Khalili Street, P.O. Box: 7193711351, Shiraz, Iran

**Keywords:** Genetic engineering, Stem cells, Cell therapy, Cirrhosis, Liver disease

## Abstract

Liver damage caused by toxicity can lead to various severe conditions, such as acute liver failure (ALF), fibrogenesis, and cirrhosis. Among these, liver cirrhosis (LC) is recognized as the leading cause of liver-related deaths globally. Unfortunately, patients with progressive cirrhosis are often on a waiting list, with limited donor organs, postoperative complications, immune system side effects, and high financial costs being some of the factors restricting transplantation. Although the liver has some capacity for self-renewal due to the presence of stem cells, it is usually insufficient to prevent the progression of LC and ALF. One potential therapeutic approach to improving liver function is the transplantation of gene-engineered stem cells. Several types of mesenchymal stem cells from various sources have been suggested for stem cell therapy for liver disease. Genetic engineering is an effective strategy that enhances the regenerative potential of stem cells by releasing growth factors and cytokines. In this review, we primarily focus on the genetic engineering of stem cells to improve their ability to treat damaged liver function. We also recommend further research into accurate treatment methods that involve safe gene modification and long-term follow-up of patients to increase the effectiveness and reliability of these therapeutic strategies.

## Introduction

Toxins in the liver can lead to acute liver failure (ALF) or progress to chronic liver disease and cirrhosis. Liver cirrhosis (LC) is the main cause of worldwide liver-related mortality [1]. It causes irreversible liver damage with loss of hepatocytes and has limited therapeutic authority [2]. Infection by viruses, drugs, autoimmune disorders, sinusoidal obstruction syndrome [3], genetic diseases, chronic alcohol abuse, and obesity are significant causes of LC [4]. Most patients in the first stage of liver disease do not show any symptoms. In these cases, advanced LC, the final pathological pathway of liver disease, occurs if essential care is not taken. Eventually, liver transplantation is the last treatment for LC disease.

This treatment has limitations because of a shortage of donor's livers, postoperative problems, immune-suppression side effects, and expensive health system governance services [5, 6]. Therefore, finding new unrestricted techniques with spectacular results is urgently needed.

Several cellular and molecular factors contribute to ALF and cirrhosis progression. Hepatocytes are frequently involved in liver regeneration; hepatic stellate cells (HSCs) generate collagen and another extracellular matrix (ECM); sinusoidal endothelial cells are present as the agents of defenestration and development of capillaries, Kupffer cells stimulate the activation of HSCs, and they destroy the hepatocytes, which contribute to the progression of LC. In the case of cirrhosis, molecular factors such as cytokines mediate complex signaling pathways that have anti-oxidative, anti-inflammatory, and anti-apoptotic effects. MiRNAs also play a significant role in cirrhosis by regulating the transcription and translation of several genes [7, 8]. The liver has an intrinsic renewal capacity [9]. But in patients with the end stages of liver disease, local stem cells could not regenerate the whole damaged tissue. So, administration of engineered stem cells can be a therapeutic option with enhanced function.

According to recent studies, stem cells have been promised as a new strategy for improving liver function [10, 12]. Commonly, two mechanisms are proposed for stem cells: the paracrine effect, which enhances the de novo generation of hepatocytes (8).

The curative properties of many types of stem cells have been investigated in LC conditions [13, 14].

As hepatocyte-like cells (HLCs) are known to contribute to the remodeling of the cirrhosis liver, some kinds of cells, such as mesenchymal stem cells (MSCs), hematopoietic stem cells (HSCs), and endothelial progenitor cells (EPCs) that have differentiation potential into HLCs, can promote liver disease [15–17]. Therefore, various sources of mesenchymal stem cells, because of their beneficial attributes, have been suggested for stem cell therapy of liver disease [18].

Other kinds of stem cells, like induced pluripotent stem cells [19] and embryonic stem cells (ESCs), can differentiate into HLCs [20].

**Fig. 1:**
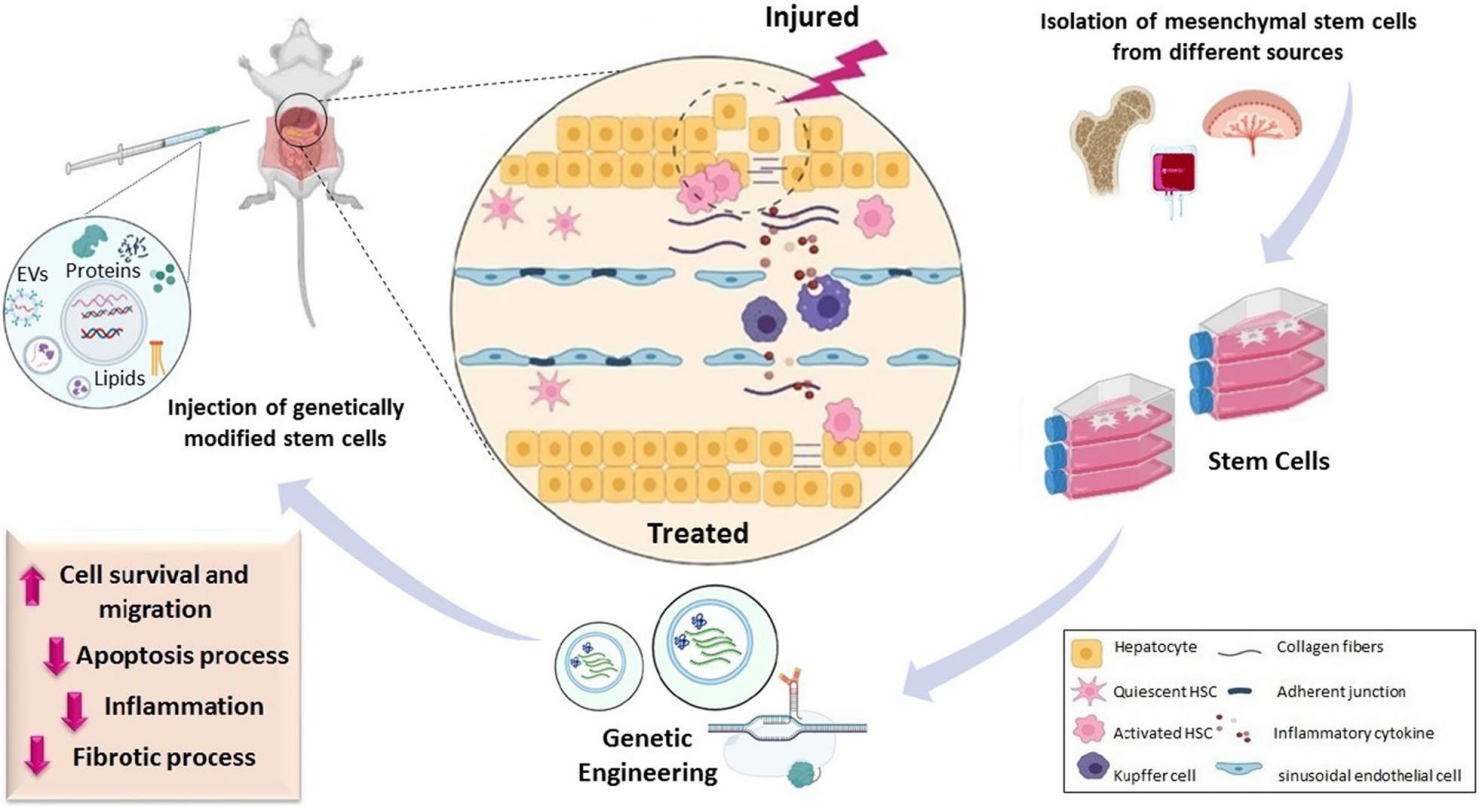
Therapeutic potential of genetically modified stem cells in liver injuries. MSCs can originate from several tissues such as umbilical cord, bone marrow, adipose, peripheral blood, etc. Genetic manipulation increases regenerative capacities of MSCs in liver diseases. Injection of engineered MSCs can attenuate activation of HSCs, collagen deposition, inflammation, apoptosis and fibrotic processes. MSCs, mesenchymal stem cells; HSCs, hepatic stellate cells

Stem cells' regenerative abilities, due to the release of growth factors, chemokines, cytokines, microRNAs, and exosomes, make them one of the best choices for cell-based therapy [21]. So, gene engineering of stem cells is a strategy to improve their natural function and therapeutic potential. In pre-clinical experiments, different types of genetically modified stem cells, pretreated stem cells, and cell-free therapy were investigated last year. In this review, we discussed the current literature according to the different approaches (Table 1) and the future outlooks of gene-engineered stem cell-based therapy in liver disease, as shown in Fig. 1.

**Table 1:** Gene modification of stem cells in the treatment of liver diseases

References	Result	Experimental model	Animal model	Administration method	Gene editing method	Dosage	Cell source	Gene
Ma et al.	Better homing of MSCs, improve hepatocyte proliferation via hepatocyte-generating factors (HGF) and vascular endothelial growth factor (VEGF), leading to reduced mortality and improved liver regeneration	Acute liver failure	Mice	Intravenously	Lentiviral transduction	1 × 10^6^	Bone marrow	CXCR-4
Wang et al.	Reducing hepatic activity index (HAI) scores	Acute liver failure	Rat	Intravenously	Lentiviral transduction	1 × 10^5^	Bone marrow	C-Met
Ma et al.	Upregulated expression of platelet‐derived growth factor D, promoting angiogenesis	Fibrosis	Rat	Intravenously	Adenovirus transfection system	400 µg of protein	Exosome-umbilical cord	AKT
Jin et al.	Increasing mRNA and protein levels of ALB, CK18, and HNF4a Better survival, and enhancing the differentiation into hepatocytes-like cells	Cirrhosis	Rat	Intravenously	Adeno-associated virus	1 × 10^5^	Bone marrow	BCL2
Zhang et a.l	Increasing protein and mRNA levels of hepatocyte nuclear factor 4α	Cirrhosis	Rat	Intravenously	Adenovirus transfection system	1 × 10^6^	Bone marrow	HGF
Tang et al.	Downregulating Bax and TNFα Upregulating Bcl2	Acute liver failure	Mice	Intravenously	Adenovirus transfection system	1 × 10^6^	Umbilical cord	HGF
Wang et al.	Increasing PCNA and EpCAM, ameliorating engraftment	Cirrhosis	Rat	Intravenously	Lentiviral transduction		Bone marrow	FGF
Fiore et al.	Upregulating PCNA mRNA, Reducing collagen deposition	Fibrosis	Mice	Intravenously	Adenovirus transfection system	5 × 105	Bone marrow	IGF-I
Fiore et al.	Increasing hMø, anti-fibrotic activities Downregulating pro-inflammatory markers expression	Fibrosis	Mice	Intravenously	Adenovirus transfection system	5 × 105	Bone marrow	IGF-I
Kim et al.	Improving ATP production, mitochondrial biogenesis, and metabolism Better engraftment into damaged area	Cirrhosis	Rat	Intravenously	Lentiviral transduction	2 × 106	Placenta	PRL-1
Zheng et al.	Downregulating pro-inflammatory cytokines Better MSCs migration and differentiation Preventing apoptosis	Fulminant hepatic failure	Rat	Intravenously	Lentiviral transduction	1 × 10^5^	Amniotic fluid	IL-1RA
Ye et al.	Enhancing nitric oxide synthase expression Improving the anti-inflammatory properties	Cirrhosis	Mice	Intravenously	Adenovirus transfection system	1 × 10^6^	Bone marrow	HNF4α
Yu et al.	Increasing IL-10 secretion levels, advancing polarization of Kupffer cells to the M2 phenotype Decreasing TNF-α and IL-1β levels	Acute liver failure	Mice	Intravenously	Lentiviral transduction	2 × 10 6	umbilical cord	HNF4α
Ma et al.	Decrease in the ratio of CXCL1, IL-1β, and IL-6 Improving liver regeneration, survival rate, and fewer inflammatory cytokines	Acute liver failure	Mice	Intravenously	Lentiviral transduction	1 × 10^6^	Bone marrow	IL-1β
Su et al.	Reducing TGF-β1, α-SMA, TIMP-1, TGFBR1, laminin and hyaluronic acid mRNA level Diminishing collagen I, III	Cirrhosis	Mice	Intravenously	Lentiviral transduction	1 × 10^6^	Bone marrow	SMAD7

## Common gene editing methods

Gene therapy involves introducing genetic material into target cells using non-viral or viral vehicles. This genetic material is used to treat or prevent diseases by replacing or repairing damaged genes. Viruses are the most common method for exogenous transgene overexpression and external genetic manipulations. Gene therapy practices ex vivo and/or in vivo techniques to produce benefits. During ex vivo gene editing, target cells such as stem cells are taken, genetically modified, and injected into the patient [12]. Several gene delivery techniques have been used to treat various disorders like liver diseases. It has been investigated whether defective genes can be replaced using gene-editing technologies such as clustered regularly interspaced short palindromic repeats-CRISPR-associated protein 9 (CRISPR-Cas9), zinc finger nucleases (ZFNs), transcription activator-like effector nucleases (TALENs), and mega-nucleases has been investigated [13]. In recent years, viral vector-mediated gene therapy has been used in pre-clinical tests and clinical trials to treat various diseases. Adenovirus, adeno-associated virus (AAV), retroviral, and lentiviral vectors are the most efficient viral vectors, as reported in several studies [14, 15]. The first gene therapy product, based on an AAV gene delivery strategy, was approved in Europe for lipoprotein lipase deficiencies in 2012 [16]. After that, numerous viral vector drugs were approved by the US Food and Drug Administration (FDA) [17–19]. Furthermore, the FDA predicted that by 2025, 10–20 new cell and gene therapy products would be authorized annually. But an efficient drug based on genetically modified stem cells has not yet been tested in clinical studies.

## Modification of genes rolled in cell survival and migration

### C-X-C chemokine receptor type 4 (CXCR-4)

Stromal-derived factor 1 alpha (SDF1α), also known as CXCL12, is the ligand of the CXC family, bound to C-X-C chemokine receptor type 4 (CXCR-4) that is expressed throughout bone marrow stromal cells. Several studies have shown essential roles for the CXCL12–CXCR4 axis in the survival, homing, and improved colonization of stem cells. Downstream signaling pathways of CXCR4 can control cell proliferation and movement via PI3K-Akt and mTORC signaling [22, 23]. In addition, CXCL12 can activate the migration of stem cells from bone marrow to destroyed organs such as the liver, lung, heart, and brain through the chemoattraction of CXCR4 on stem cell membranes. It has been suggested that CXCL12–CXCR4 can enhance stem cell transplantation, which is necessary to regenerate damaged tissue [24–26]. Indeed, MSCs express *CXCR4* at low levels, so overexpression of *CXCR4* seems advantageous to improve organ functions.

Ma et al. [22] injected modified *CXCR4* gene MSCs with lentiviral transduction and null-MSCs intravenously into nude mice a day after chemical carbon tetrachloride (CCL4) administration. They indicated that after ALF, the concentration of CXCL12 was increased. Furthermore, in vivo imaging techniques illustrated that CXCR4-MSCs mobilized more than null-MSCs to the damaged liver. Results demonstrated that genetically modified cells enhanced the homing of MSCs. So, in the following analysis, Ki-67 immunohistochemical assays showed raised cell proliferation and levels of hepatocyte-generating factors (HGF) and vascular endothelial growth factor (VEGF) and better liver function.

### Tyrosine-protein kinase Met (c-Met) or hepatocyte growth factor receptor

Several studies [27–29] found that hepatocyte growth factor (HGF), a motility and trophic factor secreted by MSCs, protects against liver damage. HGF is the ligand of c-Met, a tyrosine kinase receptor family member. The HGF/c-Met axis is crucial in the proliferation, regeneration, development, protection, scattering process, and differentiation of BMSCs into hepatocytes [27, 30–33]. However, the insufficient capacity of stem cells to reside in the damaged liver has been a concern for their therapeutic properties.

Wang and his colleagues [34] overexpressed c-Met protein in BMSCs using lenti-*C-Met*-GFP vectors (*C-Met*-BMSCs). In vitro assays demonstrated the increased migration activity of c-Met-BMSCs against the control BMSC group associated with HGF. This study showed that improving the homing of BMSCs through increased cell surveillance and decreasing the hepatic activity index (HAI) scores could all facilitate repairing the ALF rat model.

In a similar study, Liu et al. [35] investigated whether HGF/c-Met signaling effectively promoted MSC migration and regeneration of the injured liver induced by intestinal ischemia–reperfusion in rats.

### Akt

A serine/threonine kinase, Akt, is well-known as protein kinase B, which has crucial roles in cellular processes like proliferation, migration, angiogenesis [36], and anti-apoptosis. As a member of the Bcl2 family, BAD is phosphorylated by Akt and reduces its pro-apoptotic property [37]. The Akt family has three different isoforms: Akt1, Akt2, and Akt3, which are encoded by distinct genes.

Despite the beneficial effect of MSCs in diverse regeneration aspects, a low survival rate in apoptosis sites has been a restrictive factor. To improve the viability of transplanted cells, Zhou et al. [38] suggested genetic modification of BMSCs with the *AKT1* gene in concanavalin A (ConA) in the injured liver of a C57BL/6 mouse. In vitro and in vivo analysis of *AKT1*-BMSCs have illustrated more viability and better homing capacity than the control group. Notably, *AKT1*-BMSCs treated mice produced lower levels of ALT, AST, TNF-α, IFN-γ, and higher concentrations of VEGF, HGF, and immunosuppressive factors like IL-10 in serum and injured liver.

Ma et al. [39] evaluated the therapeutic effect of exosomes derived from *AKT* gene-modified human umbilical cord mesenchymal stem cells (Akt-Exo). They reported that Akt-Exo significantly upregulated the expression of PDGF, which caused an improvement in the proliferation, migration, and angiogenesis of endothelial cells.

## Gene modification of trophic factors

### Hepatocyte growth factor (HGF)

HGF is a liver-regenerative factor that is elevated during liver injuries and hepatectomy [48–50]. HGF has been identified as a potent multifunctional cytokine that can stimulate mitogenesis, morphogenesis, cell growth, differentiation, and motility [51, 52]. Subsequent HGF binding to its specific receptor, c-Met, initiates a signaling cascade of critical biological actions like development, homeostasis, and regeneration [50, 53, 54]. So, the HGF/c-Met signaling pathway is considered to regulate liver damage.

Zhang et al. [55] investigated the protective effect of BM-MSCs genetically engineered with *HGF* with an adenoviral vector containing a green fluorescent protein (EGFP) label (*HGF*-BM-MSC group). Different groups were transplanted intravenously in the rat cirrhosis model induced by CCL4. After 4 weeks of treatment, the data analysis showed that the HGF-BM-MSC group enhanced the mRNA and protein expression levels of HNF-4α, CK18, and ALB in the liver. On the other hand, some liver injury markers, such as aspartate aminotransferase (AST), alanine aminotransferase (ALT), and total bilirubin (TBIL), showed significant elevation in the serological test. Due to the exogenous HGF’s short half-life, HGF-BM-MSC seems affordable for treating liver cirrhosis and disease.

In another experiment, Tang et al. [56] discovered the therapeutic potential of human umbilical cord-derived mesenchymal stem cells (UCMSCs) that overexpress HGF in the ALF mouse model. Their results indicated that HGF-UCMSCs could gain the activity of *γ*-glutamyl cysteine synthetase (*γ*-GCS), superoxide dismutase (SOD), and catalase (CAT), which are involved in cellular redox homeostasis. Additionally, this group had anti-apoptotic features by downregulating Bax and TNFα and upregulating *BCL-2* genes.

### Fibroblast growth factor (FGF)

Fibroblast growth factors (FGFs) comprise a large family of cell signaling proteins. This growth factor is highly conserved in gene and amino acid sequencing between vertebrates [57]. The FGF family is composed of 22 ligands that have their special tyrosine kinase receptors (FGFRs). The FGF/FGFR signaling pathway can regulate biological processes like cell survival, proliferation, differentiation, migration, embryonic development, organogenesis, metabolism, and regeneration [58].

A previous cirrhosis liver study reported that FGF4 can induce BMSCs into HLCs, which indicated that this differentiation might be the effect of secreted cytokines from damaged liver cells [59]. Wang et al. [60] modified BMSCs with a recombinant FGF4 lentiviral vector and found that FGF4-BMSC improved BMSC engraftment in cirrhotic liver rats. Furthermore, compared to another group, they observed more proliferating cell nuclear antigen (PCNA), Jagged-1, and epithelial cell adhesion molecule (EpCAM)-positive hepatocytes. These results suggest that *FGF-4*-modified BMSCs might be involved in liver regeneration by ameliorating engraftment and proliferation of BMSCs and modulating cirrhotic liver cell microenvironments.

## Gene modification enrolled in the anti-apoptosis process

### B-cell lymphoma 2 (Bcl2)

Apoptosis is a crucial biological process for tissue development and homeostasis. This programmed cell death procedure mediates hepatic cirrhosis and influences the liver’s regenerative ability [40, 41]. The mechanism of apoptosis occurs via two main pathways; one is the extrinsic way, which is found by the interaction between a ligand and tumor necrosis factor (TNF) death receptors. Death-inducing signaling complexes then bind to their adaptors and lead to activation of caspase-8, caspase-3 cascades, and finally, cell death [42, 43]. The other is an intrinsic way that is caused by endogenous cellular stresses like metabolic disturbances, growth factor deprivation, DNA damage, and oxidative stress. Then, due to the intrinsic pathway, mitochondrial depolarization and cytochrome c release occur. Cytochrome c can bind to apoptosis protease-activating factor 1 (APAF1) and procaspase-9, assembling an apoptosome that triggers the activation of caspase-9. Therefore, downstream caspase-3, caspase-7, and caspase-6 are activated [43]. The Bcl-2 family of proteins controls the critical activity of the intrinsic apoptotic pathway by regulating mitochondrial outer membrane permeabilization (MOMP) [44]. In several research studies [45, 46], the *BCL-2* gene has been known as an apoptosis repressor gene.

Jin and co-workers [47] transplanted BMSCs with *BCL-2* overexpression into cirrhotic rats induced by CCL4. Expression of albumin (*ALB*), cytokeratin 18 (*CK18*), and hepatocyte nuclear factor 4a (*HNF4a*) was examined in HLCs, which were integrated with adeno-associated virus (AAV) as a vector for overexpressing the *BCL-2* gene (AAV-*BCL-2*). The BMSCs-AAV-*BCL-2* cirrhosis group indicated the highest mRNA level and hepatocyte markers, such as ALB, CK18, and HNF4a, on day 28. Finally, they reported that genetic modification of BMSCs with the *BCL-2* gene enhanced cell survival, differentiation to HLCs, and recovered liver function in cirrhotic rat models.

### Phosphatase of regenerating liver-1 (PRL-1)

Phosphatase of regenerating liver-1 (PRL-1), as a member of the PRL family, is a tyrosine phosphatase and primary response gene in liver cell repair [65]. Although the PRL‐1 mRNA expression value varies in different tissues, higher levels of PRL-1 have been found in growing hepatic cells. The early growth response protein 1 (Egr‐1) transcription factor induces *PRL‐1* expression in liver regeneration [66, 67]. PRL-1 can regulate cell proliferation and differentiation and affect the migration and invasion processes by promoting matrix metalloproteinase (MMP)-2 and MMP-9 expression via the proto-oncogene c-Src and ERK1/2 pathways [68–73]. Jiao et al. [74] reported that PRL-1 is necessary for the normal timing of cell cycle progression within liver regeneration and has an anti-apoptotic effect.

Kim et al. [75] generated placenta-derived mesenchymal stem cells (PD-MSCs) overexpressing *PRL-1* (PD-MSCs^PRL-1^) to analyze their performance in rat liver cirrhosis induced by administration of bile duct ligation (BDL) for 10 days. Outcomes showed that enhanced *PRL-1* expression in PD-MSCs could improve ATP production, mitochondrial biogenesis, and metabolism, better engraft into damaged areas, and eventually accelerate liver function through mitochondrial dynamics.

### Interleukin-1 receptor antagonist (IL-1RA)

Another member of the IL-1 family of cytokines is IL-1Ra, which inhibits the pro-inflammatory activity of IL-1α and IL-1β and modulates their immune and inflammatory reactions [76, 77]. IL-1Ra can bind to the IL-1 receptor and prevent signal transduction into the cell. As a result, the natural equivalence of IL-1 and IL-1Ra is critical and mediates inflammatory events [78, 79]. Previous findings report that IL-1Ra can prevent apoptosis [80] and have hepatoprotective effects [81].

Zheng et al. [82] investigated *IL-1Ra* overexpression in amniotic fluid-derived mesenchymal stem cells (*IL-1Ra*-AF-MSCs) in a rat model of fulminant hepatic failure (FHF). The implantation of *IL-1Ra*-AF-MSCs into the damaged liver via the portal vein resulted in the downregulation of pro-inflammatory cytokines such as IL-1, IL-6, and TNF-α; improved MSC migration rates; higher potential in hepatic differentiations; the prevention of hepatocyte apoptosis; and significant liver function.

## Gene modification plays a role in the anti-inflammatory properties

### Hepatocyte nuclear factor-4 alpha (HNF4α)

HNF-4 is a nuclear transcription factor that controls the morphogenesis and maturation of liver cells and is known as a consequential regulator of hepatocyte differentiation [83, 84]. Furthermore, regular *HNF-4* expression can restore hepatocyte function [85, 86]. It was reported that *HNF-4α* has less expression in liver disease conditions; therefore, researchers suggested overexpression of *HNF-4α* can increase the curative effect of this factor on liver damage [86].

Ye et al. [87] used CCL4 to induce a liver cirrhosis model. Three weeks after the induction of cirrhosis, MSCs modified by *HNF-4α* overexpression adenoviruses (*HNF-4α*-MSCs) were injected into the mice's tail vein. The result showed that HNF-4α could improve the anti-inflammatory properties of MSCs by enhancing nitric oxide synthase (iNOS) expression by activating the NF-κB signaling pathway.

Yu et al. [88] administered human umbilical cord mesenchymal stem cells with overexpressed *HNF4α* (HuMSC-*HNF4α*) to mice with ALF induced by D-galactosamine/lipopolysaccharide (D-galN/LPS). They discovered that HuMSC-*HNF4* promoted Kupffer cell polarization to the M2 phenotype, inhibited macrophage inflammatory responses by secreting higher levels of IL-10 and macrophage colony-stimulating factor (M-CSF), and reduced the expression of inflammatory factors such as TNF- and IL-1, which inhibited inflammation and regenerated injuries.

### Interleukin 1 beta (IL-1β)

IL-1β is a primary cytokine that activates immune and inflammatory responses encoded by the *IL1B* gene. Following tissue injury, activated macrophages produce excessive IL1 and recruit inflammatory cells [89–91]. Small interfering RNAs (siRNA) benefit from high target selectivity and low toxicity and can help regulate inflammatory responses. Ma et al. [92] prepared MSC combined with *IL-1β* siRNA adenovirus for implantation into the tail vein of the ALF mouse model. The results showed notably reduced levels of CXCL1, IL-1β, and IL-6 as the primary inflammation cytokines by MSC + *IL-1β* siRNA treatment, and ALT and AST levels changed significantly compared to the control group. In addition, models treated with MSC and *IL-1* siRNA had better liver regeneration, higher survival rates, and lower inflammatory cytokines, indicating an effective ALF strategy.

## Gene modification role in the anti-fibrotic process

### Insulin growth factor-I (IGF-I)

Insulin growth factor I (IGF-I) is a hormone produced by the liver and is involved in anabolic reactions. Experiments demonstrated this hormone level had decreased in the cirrhotic liver [61]. IGF-I can stimulate cell growth and metabolic pathways [62].

Fiore et al. [63] aimed to study the efficacy of *IGF-I* overexpressing BMSCs on fibrotic liver mice. A day after genetically modified MSCs to express *IGF-I* (Ad*IGF-I*-MSCs) transplantation, PCNA mRNA values were upregulated, especially in hepatocytes. Also, collagen deposition reduction and suppression immunogenicity against adenoviral antigens were elucidated in this group.

In another study, Fiore and his colleagues [64] found that hepatic macrophage (hMø) numbers increased in Ad*IGF*-*I*-MSCs-treated fibrosis mice and demonstrated a reduced pro-inflammatory and pro-fibrogenic gene expression profile and decreased oxidative stress levels. Furthermore, expression profile analyses showed downregulation of pro-inflammatory markers and significant gene regulation in the DNA repair and synthesis cell cycle. In addition, they reported that hMø participated in AdIGF-I-MSCs anti-fibrotic activities. After being treated with Ad*IGF-I*-MSCs, fibrotic livers had expression profile analyses for cell cycle markers performed on them. The profiles showed significant gene regulation related to DNA synthesis and repair quality control, cell cycle progression, and DNA damage/cellular stress compatible with the early induction of pro-regenerative and hepatoprotective mechanisms.

### Mothers against decapentaplegic homolog 7 (Smad7)

Smad7 is a member of the Smad family that regulates transforming growth factor (TGF-β) signaling [93, 94]. TGF-β ligands stimulate the Smad-2/3 pathway and the expression of several profibrotic genes, including various types of collagens [95, 96], plasminogen activator inhibitor-1 (*PAI-1)* [97, 98], integrins [99], some proteoglycans [100, 101], MMPs [102], and connective tissue growth factor [103]. Smad7 is a negative modulator of TGF-β [104]. Wu et al. discovered that increased *SMAD7* gene expression in rat MSCs could prevent fibrogenesis in HSCs [105].

Su et al. [106] investigated the curative potential of genetically modified MSCs overexpressing the *SMAD7* gene in an 8-week CCL4-induced cirrhosis liver rat model. 7 and 21 days after injection of Smad7-MSCs into the main lobes of the cirrhotic liver, both protein and mRNA values of Smad7 were increased. Treatment with Smad7-MSCs diminished the serum levels of collagen I, III, and collagenase I, III. This approach caused a reduction in the mRNA levels of TGF-β1, α-SMA, TIMP-1, TGFBR1, laminin, and hyaluronic acid. In this in vivo study, cell-based gene therapy was applied to improve cirrhosis liver function by inhibiting TGF-β1 signaling.

## microRNAs manipulation

MicroRNAs (miRNAs) are short non-coding RNAs that can control gene expression at the posttranscriptional or translational levels [107]. miRNAs involve various biological processes, like proliferation, differentiation, immune responses, apoptosis, tumorigenesis, and tissue remodeling [108, 109]. Recent research suggests that miRNAs play a role in liver regeneration and could be a therapeutic strategy in liver disease [110, 111].

Qu et al. [112] showed the anti-fibrotic effects of exosomes derived from miRNA‐181‐5p overexpressed adipose‐derived mesenchymal stem cells (ADMSCs) in the CCL4‐induced liver fibrosis mouse model. In addition, exosomes containing miR181‐5p downregulated *STAT3* and *BCL-2* expression and activated autophagy, which revealed the reduction in extracellular matrix components.

In another study on ALF, Liu's team [113] used exosomes isolated from miR-17-knockdown adipose tissue-derived MSCs (AMSC-Exo^miR-17-KD^) to find the role of miR-17 in AMSC-Exo-based therapy. They determined miR-17 can target thioredoxin-interacting protein (TXNIP) and inhibit nucleotide-binding and oligomerization domain-like receptor 3 (NLRP3) inflammasome activation in macrophages. NLRP3 is significantly expressed in the liver and plays a role in fibrosis. Lou et al. [114] identified a new potential approach for improving liver fibrosis by administering MiR‐122‐modified AMSCs. Data analysis showed serum markers such as hyaluronic acid (HA), procollagen III‐N‐peptide (P‐III‐P), ALT, and decreased AST levels. In addition, the expression levels of TGF‐β1 and α‐SMA notably downregulated, and, importantly, MiR122‐ AMSCs suppressed HSC proliferation and collagen maturation.

## Conclusion

Until now, stem cells, especially MSCs, have been considered remarkable applicants for regenerative medicine in liver disease due to their beneficial properties, including differentiation into hepatocyte-like cells, producing chemokine factors, immunomodulatory effects, anti-fibrotic, anti-apoptotic, and anti-oxidant activities [115]. However, there are still some challenges in clinical administration, like the genetic modification of stem cells through the manipulation of target genes, which can enhance the rate of stem cell survival and engraftment in damaged liver tissue and improve their therapeutic potential. In addition to the advantages of genetic engineering, there are still several limitations to having a functional next-generation MSC-based cell therapy. For instance, the sufficient dosage of transplanted cells, optimal timing, and injection frequency is still being determined. Furthermore, the transplantation route is unclear, and the risk of unanticipated differentiation and tumorigenicity causes safety concerns. Due to different types of liver damage, we recommend further studies on accurate treatment methods with optimal conditions, safe gene modification, and long-term follow-up of cases to increase the reliability of these therapeutic strategies.

## Data Availability

All data are available in this article.

## Abbreviations

ALF: Acute liver failure
LC: Liver cirrhosis
HSCs: Hepatic stellate cells
ECM: Extracellular matrix
HLCs: Hepatocyte-like cells
MSCs: Mesenchymal stem cells
EPCs: Endothelial progenitor cells
ESCs: Embryonic stem cells
CXCR-4: C-X-C chemokine receptor type 4
SDF1α: Stromal-derived factor 1 alpha
CCL4: Chemical carbon tetrachloride
HGF: Hepatocyte-generating factors
VEGF: Vascular endothelial growth factor
C-Met: Tyrosine-protein kinase Met
Bcl2: B-cell lymphoma 2
TNF: Tumor necrosis factor
ALB: Expression of albumin
CK18: Cytokeratin 18
HNF4a: Hepatocyte nuclear factor 4a
FGFs: Fibroblast growth factors
IGF-I: Insulin growth factor like-I
PCNA: Proliferating cell nuclear antigen
PRL-1: Phosphatase of regenerating liver-1
IL-1RA: Interleukin-1 receptor antagonist
HNF4α: Hepatocyte nuclear factor-4 alpha
IL-1β: Interleukin 1 beta
Smad7: Mothers against decapentaplegic homolog 7
